# 733. The attributable mortality, length of stay, and healthcare costs of methicillin-resistant *Staphylococcus aureus* infections in Singapore

**DOI:** 10.1093/ofid/ofad500.794

**Published:** 2023-11-27

**Authors:** Yiying Cai, Edwin Philip, Shalvi Arora, Jean X Y Sim, Sean Whiteley, Weien Chow, Daniel Tiang, Siow Leng Neo, Wei Wei Hong, Indumathi Venkatachalam, Nicholas Graves

**Affiliations:** Duke NUS Medical School, Singapore, Singapore; Singapore General Hospital, Singapore, Not Applicable, Singapore; Singapore General Hospital, Singapore, Not Applicable, Singapore; Singapore General Hospital, Singapore, Not Applicable, Singapore; Axomem, Singapore, Not Applicable, Singapore; Changi General Hospital, Singapore, Not Applicable, Singapore; Singhealth, Singapore, Not Applicable, Singapore; Singhealth, Singapore, Not Applicable, Singapore; SingHealth, Singapore, Not Applicable, Singapore; Singapore General Hospital, Singapore, Not Applicable, Singapore; Duke-NUS Medical School, Singapore, Not Applicable, Singapore

## Abstract

**Background:**

Infections caused by methicillin-resistant *Staphylococcus aureus* (MRSA) are known to compromise patient outcomes and increase healthcare costs. Previous evidence on the prolongation of length of stay due to MRSA infections in Singapore is limited by methodology, including time-dependent bias. We used a multistate model to estimate the expected attributable mortality, length of stay, and healthcare costs of MRSA infections in Singapore.

**Methods:**

We conducted a retrospective case-cohort study in patients admitted to a Singapore tertiary hospital from 2018 – 2022. Patients with MRSA infections were matched to those without infections in a 1: 3 ratio, by age and admitting specialty. We used a multistate model to derive excess length of stay (Fig 1). Attributable cost of MRSA infections from the healthcare perspective (in 2023 Singapore dollar) was estimated by multiplying excess length of stay with the monetary value of a bed-day and MRSA incidence. Cox regression with adjustment of confounders was used to derive hazard ratios (HR) for mortality estimates.

**Figure:**
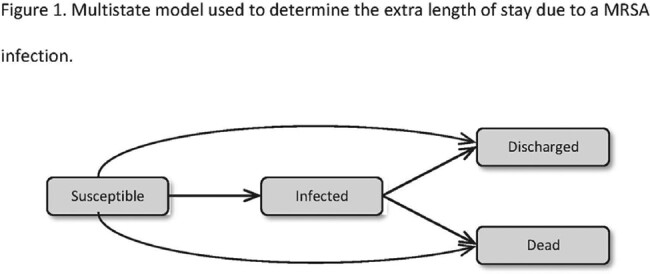

**Results:**

We matched 752 patients with MRSA infections to 2,256 patients without MRSA infections. The most common MRSA infections were skin and soft tissue infections (44.5%) and bacteraemia (18.9%). From the multistate model, the excess length of stay of a MRSA infection was 3.02 days (95% CI, 1.90 – 4.13 days). This translated to an excess healthcare cost of $594,128 (95% CI, $375,111– $813,144) per 100,000 admissions. MRSA skin and soft tissue infections had the longest length of excess stay (3.98 days; 95% CI, 2.50 – 5.46 days) and contributed most to health-care costs ($348,788 per 100,000 admissions; 95% CI, $219,099 – $478,476). After adjusting for potential confounders, the mortality risk of all MRSA infections compared to patients without infection was 1.40 (95% CI, 0.94 – 2.08). Among the different infection types, pneumonia (HR, 4.28; 95% CI, 1.25 – 14.57) and bacteraemia (HR, 2.60; 95% CI, 1.33 – 5.08) had the high mortality risk compared to patients without infection.

**Conclusion:**

Patients with MRSA pneumonia and bacteraemia had highest mortality risk. MRSA skin and soft tissue infections were associated with the highest healthcare costs. Coordinated efforts to reduce these infections may save lives and free valuable resources.

**Disclosures:**

**Sean Whiteley, BSc (IT)**, Axomem: Board Member|Axomem: Fees received for service and software licenses

